# Incidence and survival of epithelial ovarian, fallopian tube, peritoneal, and undesignated abdominal/pelvic cancers in Sweden 1960–2014: A population-based cohort study

**DOI:** 10.1186/s12885-021-08169-w

**Published:** 2021-04-26

**Authors:** Pia Leandersson, Thomas Hogberg, Paul W. Dickman, Susanne Malander, Christer Borgfeldt

**Affiliations:** 1 Reproductive Medicine Center, Skåne University Hospital Malmö, Lund University, Jan Waldenströms Gata 47, 21428 Malmö, Sweden; 2 Department of Cancer Epidemiology, Skåne University Hospital Lund, Lund University, Lund, Sweden; 3 Department of Medical Epidemiology and Biostatistics, Karolinska Institutet, Stockholm, Sweden; 4 Department of Oncology and Pathology, Skåne University Hospital Lund, Lund University, Lund, Sweden; 5 Department of Obstetrics and Gynecology, Skåne University Hospital Lund, Lund University, Lund, Sweden

**Keywords:** ovarian cancer, histopathology, long-term follow-up, relative survival, population-based, cancer registry

## Abstract

**Background:**

Despite improved surgical and oncological treatment, ovarian cancer continues to be the most lethal of the gynecologic malignancies. We aimed to analyze survival trends in epithelial ovarian cancer with regard to age, tumor site, and morphology in Sweden 1960 to 2014.

**Methods:**

A nationwide population-based study was conducted using data from the Swedish Cancer Registry on 46,350 women aged 18 or older with a diagnosis of epithelial ovarian, fallopian tube, peritoneal, or undesignated abdominal/pelvic cancer 1960 to 2014. Analyses of age-standardized incidence and relative survival (RS) were performed and time trends modelled according to age, tumor site, and morphology.

**Results:**

Overall incidence of ovarian, tubal, peritoneal, and undesignated abdominal/pelvic cancers declined since 1980. Median age at diagnosis increased. Serous carcinoma increased in incidence. RS at 1, 2 and 5 years from diagnosis improved since 1960, although not for the youngest and the oldest patients. Ten-year RS did not improve. The best RS was found for fallopian tube cancer and the worst RS for undesignated abdominal/pelvic cancer. Among the morphologic subgroups, endometrioid carcinoma had the best RS.

**Conclusions:**

Survival in epithelial ovarian, tubal, peritoneal, and undesignated abdominal/pelvic cancers in Sweden has improved over the last six decades. Advances in epithelial ovarian cancer treatment have extended life for the first 5 years from diagnosis but 10-year survival remains poor.

## Background

Epithelial ovarian, fallopian tube, peritoneal, and undesignated abdominal/pelvic cancers constitute a cluster of interrelated diagnoses. The diagnosis assigned to a specific patient depends on prevailing diagnostic and treatment algorithms. New knowledge about the origin of ovarian cancer, with a majority of epithelial ovarian cancer found to evolve from primary lesions in the fallopian tubes, has resulted in a larger proportion of fallopian tube cancer diagnoses [1, 2]. Patients with advanced cancer not eligible for surgical staging will today be diagnosed with undesignated abdominal or pelvic cancer, while in earlier time periods, a diagnosis of ovarian cancer was made in many cases without a specific biopsy from the adnexa [3]. The category of undesignated abdominal or pelvic cancer is bound to include some advanced tumors of other gynecological origin, and gastrointestinal malignancies. However, excluding this group would introduce considerable bias when comparing different time periods. A trend towards primary surgery as opposed to neoadjuvant chemotherapy in advanced cases is likely to result in a larger proportion of patients with site-specific diagnoses. There are good reasons to analyze this group of tumors together in order to get a more comprehensive picture of time trends for incidence and survival. In the following text we use the term epithelial ovarian cancer (EOC) in referring to this group.

During the last decades there have been some paradigmatic changes regarding the standard treatment for EOC. Aggressive cytoreductive surgery for advanced disease with the aim of complete tumor debulking is now considered the standard of care [4]. Primary debulking surgery followed by adjuvant chemotherapy is recommended in patients where optimal debulking is considered feasible, while patients with unresectable disease or severe comorbidity may benefit more from neoadjuvant treatment followed by interval debulking surgery [5]. Centralization to high-volume hospitals and surgeons subspecialized in gynecologic tumor surgery has improved surgical outcome and survival in ovarian cancer [6]. Since 2012, the Swedish national guidelines have recommended centralization of ovarian cancer treatment. The chemotherapy regimens have evolved since the introduction of chemotherapy for advanced ovarian cancer in the 1960s and ‘70s [7], with the most significant landmarks being the introduction of platinum in the 1980s followed by the taxanes in the late 1990s [8]. The combination of carboplatin and paclitaxel continues to be the standard therapy for EOC in Sweden today.

Survival studies of EOC display considerable variation in patient selection, making comparison of survival between populations difficult. Many do not report tumor morphology, and follow-up periods vary [9–16]. Most studies have included patients with ovarian cancer only [9, 11, 14, 16]. In later years tubal and peritoneal cancer have been included, in recognition of the common histopathological features shared by these tumors [10, 15]. By also including patients with undesignated abdominal/pelvic cancer in the current study, we aim to provide estimates of EOC survival that are more comparable over time. The only study to our knowledge to include these patients in survival analyses is the recent Swedish study by Dahm-Kähler et al. on serous EOC [13].

The aim of this study was to investigate time trends for incidence and long-term relative survival in epithelial ovarian, fallopian tube, peritoneal, and undesignated abdominal/pelvic cancers, and in later periods, morphology and FIGO (International Federation of Gynecology and Obstetrics) stage in Sweden 1960 to 2014, using data from the Swedish Cancer Registry (SCR).

## Methods

### The Swedish Cancer Registry (SCR)

The population-based nationwide SCR started registration in 1958. Coverage is secured by a mandatory requirement for health care providers (both clinicians and pathologists) to register all patients with premalignant and malignant conditions as well as certain benign tumors. Data are collected by six regional registries in close collaboration with reporting institutions in their respective regions. The completeness of registration is over 95%, and 99% of cancer cases are verified by morphology [17]. For the years 1958–1986 the site of tumors was coded in the International Classification of Diseases, Revision 7 (ICD-7), 1987–1992 in ICD-9, 1993–2004 in International Classification of Diseases for Oncology, Revision 2 (ICD-O/2), and from 2005 in ICD-O/3. For the whole period the codes have been translated into ICD-7 codes by the SCR. Information about tumor stage has been collected since 2004. Gynecologic tumors are stage-coded according to the FIGO criteria, recently revised in 2014 [3]. For the whole time period tumor morphology was coded according to the World Health Organization Statistical Code for Human Tumors (WHO/HS/CANC/24.1) (WHO 1956). A more detailed morphology coding according to ICD-O/2 was used 1993 to 2004, and since 2005 ICD-O/3 has been used. Registration of high/low grade was not included in the SCR before 2014. Serous tumors are thus analyzed as one entity in the current study. The SCR does not include information on treatment. The personal identification number used in official registries in Sweden since 1947 ensures near to complete follow-up of the patients up to the time of death or emigration. The Swedish Cause of Death Registry includes virtually all deaths since 1911, and data can be linked to other national health registries including the SCR.

### The patient cohort

**Fig. 1:**
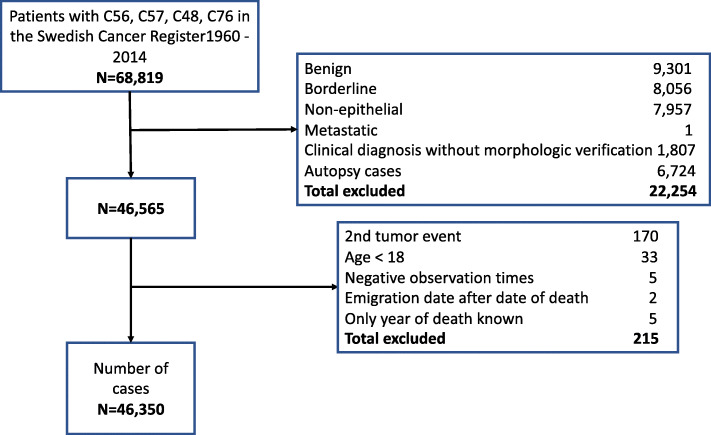
Flow chart of patients who met inclusion and exclusion criteria

A total of 465,288 cases with a diagnosis of gynecologic malignancies (ICD-7: 171–176.9, ICD-10: C51.0–C58.9), peritoneal malignancy (ICD-7: 158, ICD-10: C48.1 and C48.2), not specified abdominal or pelvic malignancies (ICD-7: 199.3 and 199.4, ICD-10 C76.2 and C76.3) registered in the SCR 1960–2014 were identified. Out of these, 68,819 cases of ovarian, tubal, peritoneal, abdominal, or pelvic malignancy were identified. Patients with benign (*n* = 9301), borderline (*n* = 8056), or non-epithelial tumors (*n* = 7957), metastatic tumor (*n* = 1), clinical diagnosis without morphological verification (*n* = 1807), and cases diagnosed incidentally at autopsy (*n* = 6724) were excluded, leaving 46,565 patients. Further, a small number of patients were excluded for the following reasons: below 18 years of age at diagnosis (*n* = 33), negative observation time (*n* = 5), date of emigration later than date of death (*n* = 2), and only date of death known (n = 5). Some cases fulfilled more than one exclusion criterion. For 170 women registered with a subsequent event of the tumors included in the study, we included only the first diagnosis in the survival analyses. We did not exclude any women on the basis of previous or subsequent diagnoses of other tumors [10]. Thus, 46,350 women aged 18 years or older with ovarian (*n* = 37,538), tubal (*n* = 1317), peritoneal (*n* = 547) and undesignated abdominal/pelvic cancer (*n* = 6948) were included in the study for analyses (Fig. 1).

Follow-up for death was available for all patients up to April 30, 2016. Survival time was calculated from date of diagnosis until date of death, date of emigration, or April 30, 2016.

### Statistics

Incidence rates were standardized to the world standard population 2011 [18]. We modelled trends in relative survival using flexible parametric models [19]. Relative survival is the ratio of overall all-cause survival for patients with the disease in question, in this case EOC, to expected survival in the general population, and can be interpreted as the survival that would be observed in a hypothetical world where it is not possible to die of causes other than EOC [20].

For the survival analyses, women were grouped into strata given their age at diagnosis (18–44, 45–54, 55–64, 65–74, and ≥ 75 years) and period of diagnosis (1960–1964, 1965–1969, … 2010–2014). We estimated relative survival within age strata along with age-standardized relative survival using the International Cancer Survival Standard population [21]. Expected mortality rates, stratified by age and calendar year, were obtained from the Human Mortality Database [22], based on data from Statistics Sweden [23]. To examine temporal trends, time since diagnosis and year of diagnosis were modelled using restricted cubic splines with 5 and 3 degrees of freedom, respectively. An explanation of the concept of cubic splines for non-linear associations in clinical practice is given in the paper by Gauthier et al. 2019 [24].

We fitted a separate model within each age group, where the effect of time since diagnosis was time varying, with 3 degrees of freedom. An illustration of the analytic approach (using publicly available data for colon cancer) is available at http://pauldickman.com/software/stata/prediction-out-of-sample/. Stata 13 (Statacorp, College Station, TX, USA) was used for the statistical analyses.

## Results

### Demography

The distributions of age, tumor site, morphology, and stage (ovarian cancer only) for the different time periods are shown in Table 1. The median age at diagnosis increased from 59 to 67 years since 1960. Stage was first included in the SCR 2004. The majority of ovarian cancer patients were diagnosed in FIGO stage III. From 2005 to 2009 to 2010–2014 the proportion of not staged patients decreased while the proportion of patients with stages III and IV increased.

**Table 1:** Demography of patient cohort

	1960–1969	1970–1979	1980–1989	1990–1999	2000–2009	2010–2014	Total
**Age median, yrs (range)**	59 (18–94)	62 (18–97)	65 (18–100)	66 (18–98)	66 (18–98)	67 (18–100)	64 (18–100)
**Age groups, yrs**	**n (%)**	**n (%)**	**n (%)**	**n (%)**	**n (%)**	**n (%)**	**n (%)**
**18–44**	806 (13)	845 (10)	842 (9.1)	690 (7.5)	514 (5.7)	204 (4.6)	3901 (8)
**45–54**	1543 (25)	1668 (20)	1345 (15)	1622 (18)	1206 (13)	531 (12)	7915 (17)
**55–64**	1836 (30)	2239 (27)	2366 (26)	2004 (22)	2364 (26)	1020 (23)	11,829 (26)
**65–74**	1454 (23)	2152 (26)	2725 (30)	2609 (28)	2370 (26)	1460 (33)	12,770 (28)
**75+**	561 (9.1)	1341 (16)	1903 (21)	2232 (24)	2633 (29)	1265 (28)	9635 (21)
**Total**	6200 (100.0)	8245 (100.0)	9181 (100.0)	9157 (100.0)	9087 (100.0)	4480 (100.0)	46,350 (100)
	**1960–1969**	**1970–1979**	**1980–1989**	**1990–1999**	**2000–2009**	**2010–2014**	**Total**
**Site**	**n (%)**	**n (%)**	**n (%)**	**n (%)**	**n (%)**	**n (%)**	**n (%)**
**Ovarian**	5560 (90)	6990 (85)	7544 (82)	7598 (83)	6899 (76)	2947 (66)	37,538 (81)
**Fallopian**	98 (1.6)	99 (1.2)	148 (1.6)	293 (3.2)	368 (4.1)	311 (6.9)	1317 (2.8)
**Peritoneal**	0 (0)	0 (0)	3 (0.03)	16 (0.17)	236 (2.6)	292 (6.5)	547 (1.2)
**Undesignated**	542 (8.7)	1156 (14)	1486 (16)	1250 (14)	1584 (17)	930 (21)	6948 (15)
**Total**	6200 (100.0)	8245 (100.0)	9181 (100.0)	9157 (100.0)	9087 (100.0)	4480 (100.0)	46,350 (100)
**Morphology**			**1995–1999**	**2000–2004**	**2005–2009**	**2010–2014**	**Total**
			**n (%)**	**n (%)**	**n (%)**	**n (%)**	**n (%)**
**Serous**			1720 (38)	1848 (39)	2144 (49)	2546 (57)	8258 (46)
**Mucinous**			455 (10)	379 (8.1)	299 (6.8)	304 (6,8)	1437 (8.0)
**Endometrioid**			535 (12)	506 (11)	393 9.0)	346 (7.7)	1780 (9.8)
**Clear cell**			192 (4.2)	164 (3.5)	180 (4.1)	197 (4.4)	733 (4.1)
**Undifferentiated**^a^			1495 (33)	1656 (35)	1234 (28)	936 (21)	5321 (29)
**Other**^b^			136 (3.0)	147 (3.1)	128 (2.9)	146 (3.2)	557 (3.1)
**N.a.**			9 (0.2)	4 (0.1)	5 (0.1)	5 (0.1)	23 (0.1)
**Total**			4542 (100.0)	4704 (100.0)	4383 (100.0)	4480 (100.0)	18,109 (100)
**FIGO stage**					**2005–2009**	**2010–2014**	**Total**
**Ovarian cancer**^**c**^					**n (%)**	**n (%)**	**n (%)**
**I**					719 (16)	774 (17)	1493 (17)
**II**					296 (6.8)	313 (7)	609 (6.9)
**III**					1619 (37)	1826 (41)	3445 (39)
**IV**					507 (12)	709 (16)	1216 (14)
**N.a.**					1242 (28)	858 (19)	2100 [24]
**Total**					4383 (100.0)	4480 (100.0)	8863 (100)

^a^Neoplasm – malignant, carcinoma NOS, carcinoma – undifferentiated NOS, carcinoma – anaplastic NOS, adenocarcinoma NOS

^b^Other morphologies: Squamous cell carcinoma NOS (81), Lymphoepithelial carcinoma (1), Transitional cell carcinoma NOS (14), Basaloid carcinoma (1), Carcinoid tumor NOS (78), Atypical carcinoid tumor (1), Signet ring cell carcinoma (17) Adenosarcoma (1), Mullerian mixed tumor (152), Mesodermal mixed tumor (39), Carcinosarcoma NOS (141), Mesonephroma malignant (31)

^**c**^Stage distribution for ovarian cancer only, due to low case load or lacking FIGO stage definitions for other tumor sites

### Incidence

**Fig. 2:**
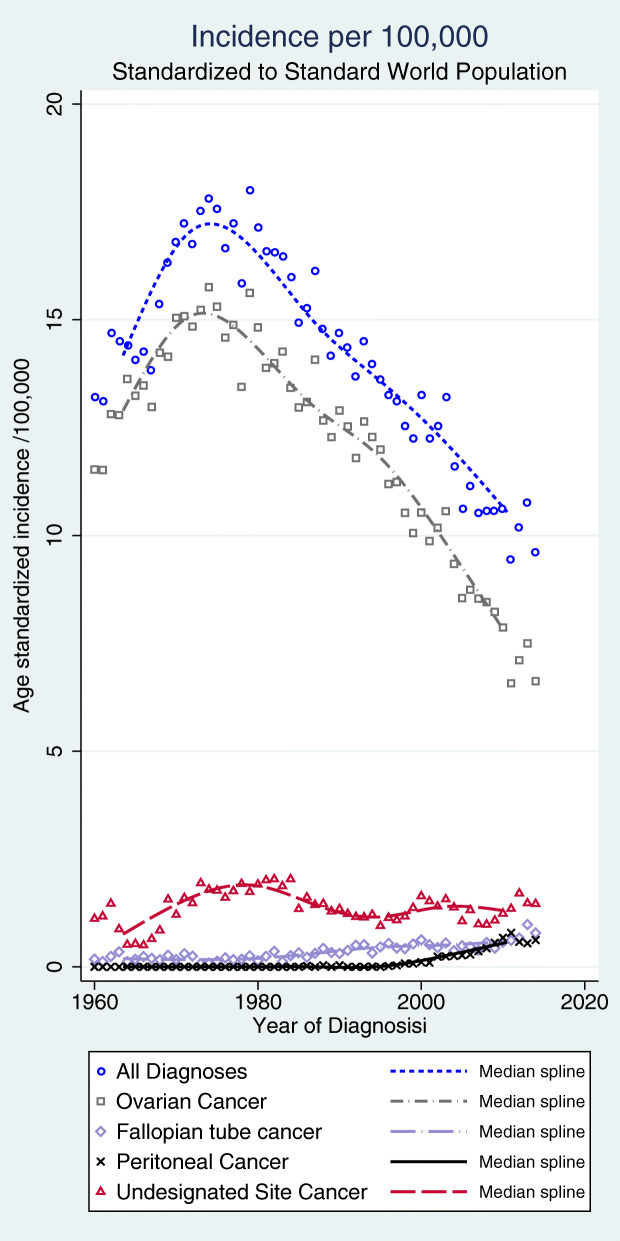
Age-standardized incidence according to tumor site per 100,000 women standardized to the world population

Since 1980 the overall age-standardized incidence of epithelial ovarian, fallopian tube, peritoneal, and undesignated abdominal/pelvic cancers together decreased markedly in Sweden (Fig. 2). The overall decrease in incidence found in this study was due exclusively to a declining ovarian cancer incidence, while fallopian tube and peritoneal cancer incidences increased. Only occasional cases of peritoneal cancer were reported before 2000.

**Fig. 3:**
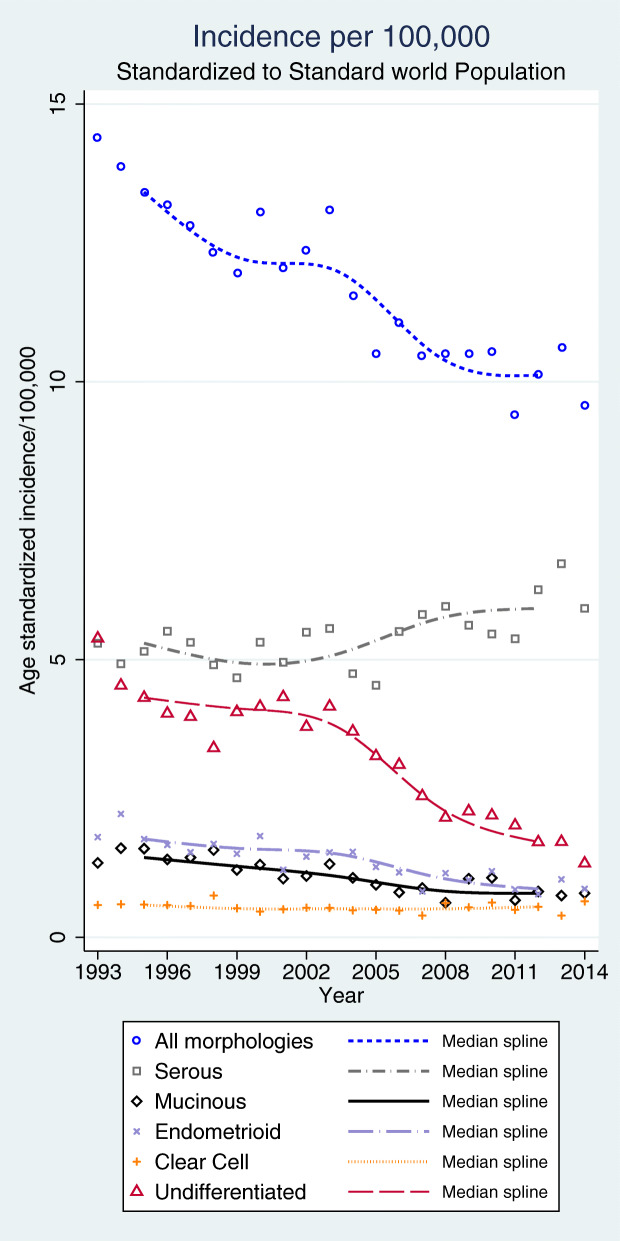
Age-standardized incidence according to morphologic subtype per 100,000 women standardized to the world population (all tumor sites)

Serous carcinomas increased in incidence 1993 to 2014, while endometrioid, mucinous, and undifferentiated carcinomas decreased (Fig. 3, all tumor sites). The incidence of clear cell cancer was stable over time.

### Relative survival (RS)

**Fig. 4:**
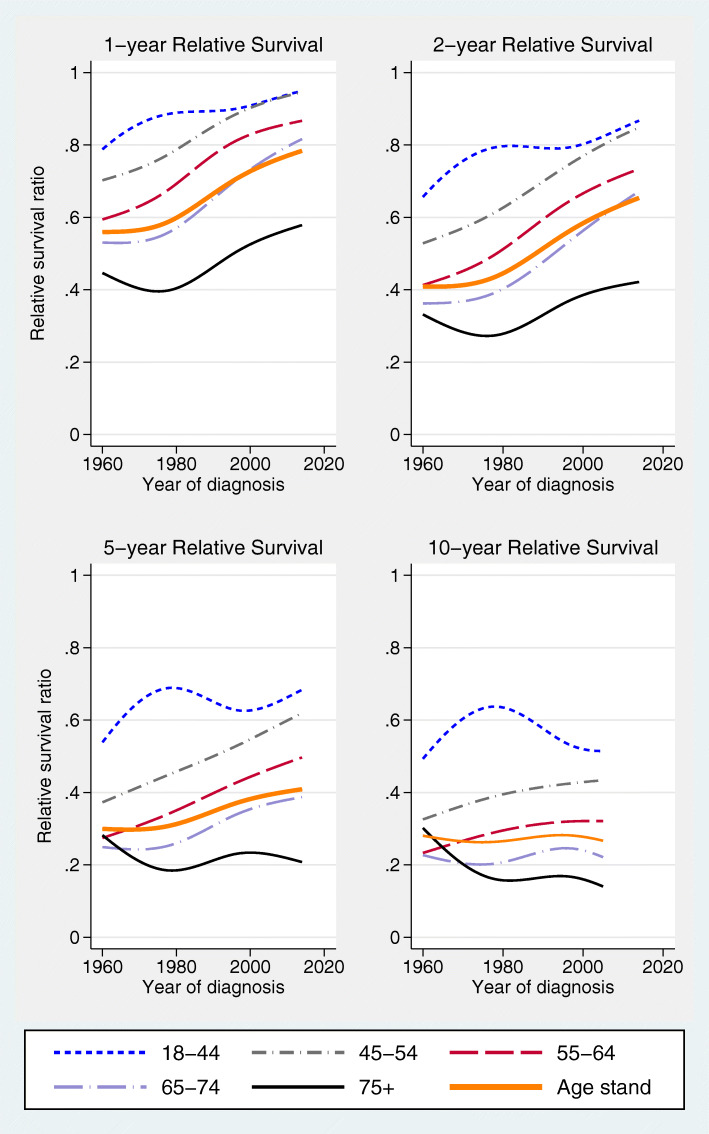
Time trends for 1-, 2-, 5-, and 10-year relative survival according to age groups and for age-standardized relative survival (all tumor sites)

A general trend of improving 1-, 2-, and 5-year age-standardized RS was found, especially since the 1980s (Fig. 4). However, this trend was not seen for 10-year age-standardized RS, which did not improve since 1960. For the youngest age group (18 to 44 years) 1-, and 2-year RS improved only slightly since 1980, converging with survival curves for the next age group (45 to 54 years) in 2000. Five-year RS did not improve since 1980, and 10-year RS declined since 1980 to the 1960 levels. The oldest women, 75+ years of age, had the worst RS. 1-, and 2-year RS improved from 1980 onwards but no improvement was found for 5-year RS and a declining trend was seen for 10-year RS.

**Fig. 5:**
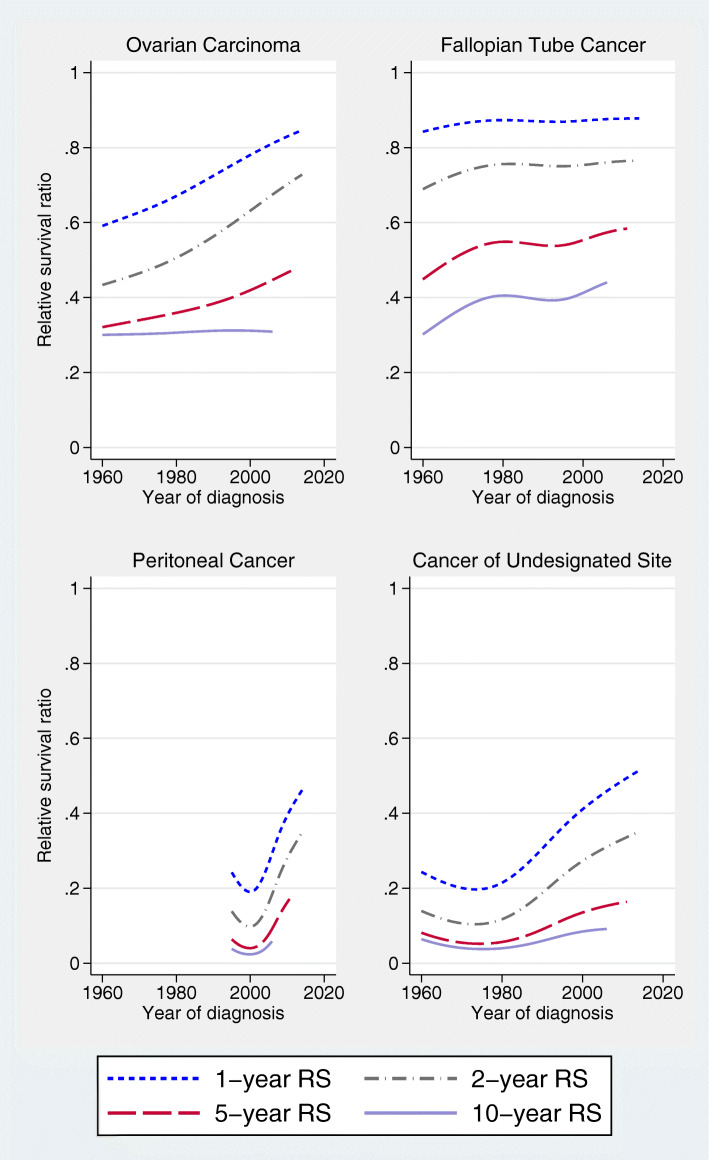
Time trends for 1-, 2-, 5-, and 10-year age-standardized relative survival according to the site of tumor

Age-standardized RS improved for all sites of tumor origin, i.e. ovarian (since 1960), undesignated abdominal/pelvic cancer (since 1980), and peritoneal cancer (since 1995) (Fig. 5). For fallopian tube cancer, RS rates increased up to 1980, with only slight improvement during subsequent decades. Very few patients were registered with fallopian tube cancer in the early time periods, and none with peritoneal cancer (Table 1). The highest RS was seen for fallopian tube cancer, and undesignated abdominal/pelvic cancer had the worst RS.

**Fig. 6:**
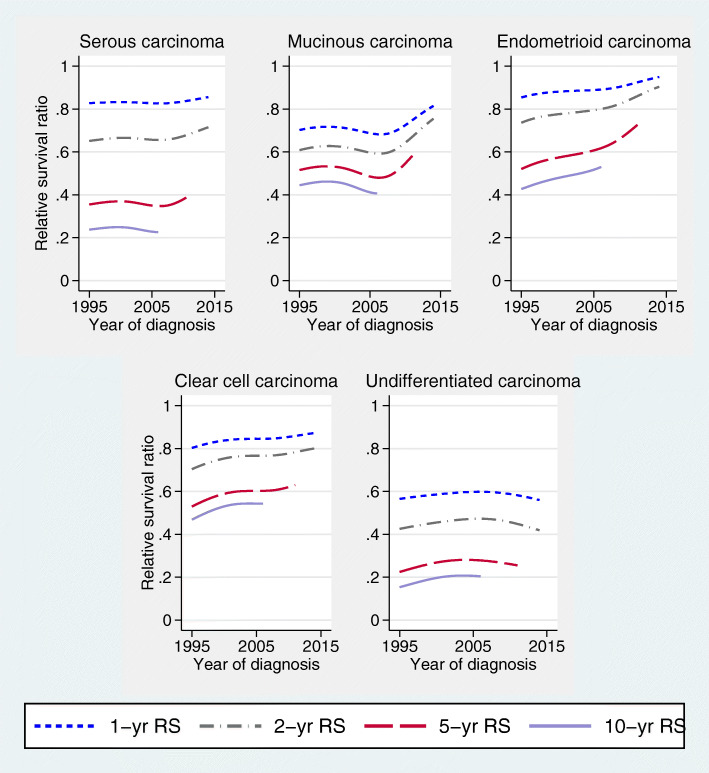
Time trends for 1-, 2-, 5-, and 10-year age-standardized relative survival according to morphologic subtype (all tumor sites)

The highest RS as well as largest improvement in RS was found for endometrioid carcinoma and the worst RS for undifferentiated carcinoma (Fig. 6). The RS rates for serous and mucinous carcinoma increased after 2007, more pronounced for mucinous carcinoma. RS improved for clear cell carcinoma since 1995. For undifferentiated carcinoma, RS rates declined after 2002.

## Discussion

### Incidence

The decline in EOC incidence found in this study has also been found in other Northern European countries and North America [10, 25]. Possible explanations for this decline have been discussed widely, with the introduction of the combined oral contraceptives in the 1960s regarded to be a major contributing factor [26]. Other well-established protective factors include parity and breastfeeding as well as tubal ligation. As mean parity has been declining in Sweden since the 1960s, temporal changes in parity and breastfeeding practices are unlikely to contribute to the decrease in EOC found in this study [23]. Tubal ligation has been common in Sweden since legalization in 1975, although in the later decades fewer women have chosen sterilization due to increased availability of safe contraceptive methods with few side effects including the levonorgestrel intrauterine system (LNG-IUS). The use of LNG-IUS has been associated with a strongly decreased risk of ovarian as well as endometrial cancer [27]. HRT (hormonal replacement therapy) has been associated with an increased risk of EOC [28]. The use of HRT has declined in Sweden as in many other countries since 2002, following the findings of the large Women’s Health Initiative randomized trial of increased risks for breast cancer and cardiovascular disease in postmenopausal women on HRT [29]. Changes in pathology classification criteria have also contributed to fewer diagnoses of EOC. Few borderline tumors were registered in the SCR in the early time periods. In the 1970s the group of borderline tumors (tumors of low malignant potential) were recognized as a separate entity in the FIGO and WHO tumor classifications and since 1960s the incidence of borderline tumors in Sweden has increased dramatically, with a steep increase in the 1980s. In the 1960s borderline tumors constituted 8% of all primary ovarian neoplasms, rising to 24% in 2000–2005 [30, 31]. Part of the decline in EOC incidence is consequently explained by a diagnostic shift from low-grade cancer to borderline tumor. In the present study we excluded borderline tumors.

Ovarian cancer incidence declined, while fallopian tube and peritoneal cancer incidences increased. The proportion of undesignated abdominal/pelvic cancer increased. Again, this reflects new diagnostic criteria, with a large proportion of the most common subtype of EOC, HGSC, now recognized to have extraovarian origin [32]. Further, in earlier time periods a diagnosis of ovarian cancer was used in many cases of disseminated disease where the ovaries could not be identified and sampled, whereas today the term “undesignated” is recommended where tumor origin cannot clearly be determined [3].

Despite the overall declining incidence in EOC, the incidence of serous carcinoma increased. There may be several explanations for this finding. Serous EOC has been found in earlier studies to increase with increasing age [25]. In the current study age at diagnosis increased during the study period, in line with an aging Swedish population [23]. The increase in serous carcinoma mirrors a decline in undifferentiated carcinoma incidence. In later decades immunohistochemistry has become a standard part of histopathological examination, adding to improved accuracy in diagnosis and consequently fewer diagnoses of undifferentiated carcinoma, and likely adding to the increased diagnosis of (high-grade) serous carcinoma. The decreasing proportion of endometrioid and mucinous carcinomas may also reflect changes in pathologic criteria. Tumors formerly classified as high-grade endometrioid are now by many pathologists regarded to be a variant of HGSC [32, 33]. A large proportion of mucinous adenocarcinoma in the ovary have been recognized as metastatic tumors, of gastrointestinal or breast origin [34]. Since the WHO tumor classification revision in 2003, more mucinous tumors formerly categorized as stage I mucinous adenocarcinoma of the ovary have been diagnosed as borderline tumors [35].

In summary, changing diagnostic classification criteria historically have caused a shift of patients between different tumor categories, partly explaining the overall decline in EOC incidence as well as declining incidences of most morphological subtypes. By including both ovarian, fallopian tube, peritoneal and undesignated primary site cancers and all morphologies in survival analyses we have aimed to minimize the impact from patients moving between tumor categories in different time periods. However, relative survival trends should be interpreted with some caution, as further discussed below.

### Relative survival

RS up to five years after diagnosis has improved during the study period, in parallel with improved surgical and oncological treatment for EOC. 10-year age-standardized RS, however, has not improved since 1960. Timmermans et al. had similar findings in their study from the Netherlands Cancer Registry of increased 5-year survival but essentially unchanged 10-year survival in EOC over 25 years [15], despite advances in cancer treatment.

There are some notable age exceptions to the overall trend of improved RS. For the youngest age group (18 to 44 years) the lack of improvement coincides with the steep increase in borderline tumor incidence since the 1980s described earlier. Since borderline tumors are more frequently diagnosed in younger women, a shift in diagnostic criteria from low-grade cancer to borderline tumor is likely to exclude a considerably higher number of patients with good prognosis from this age group compared to older age groups [30]. The poorest RS was found for the oldest women, 75 + years, in line with the findings of other EOC survival studies [9, 11, 12, 14]. Improved 1-, and 2-year RS in this age group may partly depend on much better tolerability to carboplatin-paclitaxel compared to earlier cisplatinum-based regimens in the elderly. The lack of improvement in 5-year RS and the decline in 10-year RS may be an effect of selection bias. In 1960–1964 8.5% of cases in the SCR were not morphologically verified compared to only 0.2% of cases 2010–2014 (data not shown). By excluding from the study cohort cases with clinical diagnosis only, it is likely that a larger proportion of patients with more advanced age and disease, with poorer prognosis, has been excluded from analyses in the earlier time periods.

Age-standardized RS improved for all sites of tumor origin with best RS found for fallopian tube cancer, and worst RS for undesignated abdominal/pelvic cancer. Undesignated abdominal/pelvic cancer, as discussed above, is likely to include a high proportion of patients with advanced stage cancer and comorbidity, often not eligible for primary surgery. In their study on serous EOC Dahm-Kähler et al. [13] found comparative results of poorest RS for cancer at undesignated primary site and best RS for fallopian tube cancer.

Earlier studies have showed varying results regarding RS for the different EOC morphologies [9, 14, 16]. In the current study the highest RS as well as the largest improvement in RS over time was found for endometrioid carcinoma. Endometrioid carcinoma is most often diagnosed in early stage, of low grade and responsive to chemotherapy, with a good prognosis in the majority of cases [2]. The above mentioned re-classification of high-grade endometrioid carcinoma into HGSC may add to the improved survival [32]. For undifferentiated cancer, RS rates declined since 2002. The incidence of undifferentiated cancer also declined since 2002, likely due to improved accuracy in histopathological diagnostics. Migration of patients with better prognosis (differentiated cancer) from the group of undifferentiated cancer may explain the declining RS. The RS rates for serous and mucinous cancer increased after 2007, corresponding to the introduction of more aggressive surgical approaches including upper abdominal surgery. Also, with many mucinous cancers of the ovary now recognized as metastatic tumors from breast or GI primaries, survival in mucinous EOC is expected to improve.

Serous carcinoma was the most prevalent morphologic subtype in the current study. Unfortunately, as registration of high grade/low grade was first included in the SCR 2014, in our analyses we did not have the opportunity to differentiate between LGSC and HGSC, which are today regarded as two different subtypes with very different clinical features [2]. LGSC constitutes less than 5% and HGSC around 70% of EOC according to Prat et al. 2012 [2]. Peres et al. in their cohort study on SEERS data from 2019 report LGSC in 2.6% and HGSC in 63.4% of EOC patients [16]. In our study the proportion of serous cancer is comparatively low, 46% for the whole study period 1960–2014 (57% in 2010–14), likely due to misclassification as endometrioid or undifferentiated cancer, with improved diagnostics thought to contribute to an increasing proportion of serous cancer and declining proportion of endometrioid and undifferentiated cancer in more recent time periods. HGSC is responsible for the majority of EOC deaths [32]. HGSC has, however, proved to be the subtype most responsive to chemotherapy, especially the platinum–taxane combination treatment [1]. Also, as HGSC in most cases presents with disseminated disease at diagnosis, patients with serous histology are most likely to benefit from the shift towards more extensive cytoreductive surgery from 2005 onward.

As stage was first included in the SCR 2005, we did not have the opportunity to observe potential stage-related RS changes for earlier time periods. From 2005 to 2009 to 2010–2014 the proportion of patients with unreported stage decreased and the proportion of patients with stages III and IV increased. Although the observation time is short, this may reflect the trend in tertiary ovarian cancer centers towards considering more patients with advanced disease for curative treatment, adequate staging being essential for choice of treatment.

### Strengths and limitations

The strength of our study is our nationwide SCR cohort with over six decades of longitudinal data. Reporting to the SCR is mandatory, and the coverage is high (> 95%), in combination with morphologic verification of the diagnosis in > 99% of cases [17]. The inclusion of undesignated abdominal/pelvic cancer in the survival analyses provides a more valid estimate of EOC survival over time. Limitations of our study are the lack of central pathology review and specification of grade of differentiation. Also, as the SCR does not contain information on treatment, we could not directly correlate survival trends to the prevailing surgical and oncological treatment strategies of the different time periods. Differences in diagnostic routines and improved histopathological classification criteria during the study period must be taken into consideration in the interpretation of the observed incidences and survival rates.

## Conclusion

Since 1980 the age-standardized incidence of epithelial ovarian, tubal, peritoneal, and undesignated abdominal/pelvic cancer together has declined in Sweden. The age-standardized 1-, 2-, and 5-year RS improved from 1960 to 2014, although this trend was not found in the youngest and the oldest women. The 10-year age-standardized RS did not improve in women diagnosed from 1960 to 2005. The observed improved short-term RS since 1960 can be explained by improved surgical techniques, better postoperative and advanced intensive care and more efficient and tolerable chemotherapeutical regimes together with improved supportive care during chemotherapy. Advances in treatment have prolonged life after diagnosis but long-term survival in EOC remains poor.

## Data Availability

The individual-level data maintained by the Swedish Cancer Registry used in this study are classified as sensitive personal data according to the General Data Protection Regulation (GDPR) and may not be processed or distributed except under special circumstances. The authors obtained access to the data according to the exception in GDPR that allows processing of sensitive personal data for research. Researchers interested in obtaining the raw data may apply to the Swedish National Board of Health and Welfare (the authority responsible for the Swedish Cancer Registry) in the same manner. The authors will be happy to provide Stata code that can be used to reproduce our results. The authors are also willing to perform additional analyses and provide results to third parties, provided such analyses are consistent with the aims of our research and the results do not enable individuals to be identified.

## Abbreviations

EOC: Epithelial ovarian cancer (ovarian, fallopian tube, peritoneal cancer, and abdominal/pelvic cancer)
FIGO: Fédération Internationale de Gynécologie et d’Obstétrique / International Federation of Gynecology and Obstetrics
HGSC: High-grade serous cancer.
HRT: Hormonal replacement therapy.
ICD: International classification of diseases.
LGSC: Low-grade serous cancer.
RS: Relative survival.
SCR: Swedish Cancer Registry.
WHO: World Health Organization.
